# In Vitro Comparison of Gutta-Percha Removal with H-File and ProTaper with or without Chloroform

**Published:** 2013-01-20

**Authors:** Zohreh Khalilak, Mehdi Vatanpour, Bahareh Dadresanfar, Pouneh Moshkelgosha, HamidReza Nourbakhsh

**Affiliations:** 1 Department of Endodontics, Dental Branch, Islamic Azad University, Tehran, Iran; 2 Private Practice, Tehran, Iran

**Keywords:** Chloroform, Endodontic, Gutta-percha, H-file, NiTi rotary system, Retreatment

## Abstract

**Introduction:**

Removal of root filling materials is one of the key steps in success of root canal retreatment. The purpose of this study was to evaluate the efficacy of H-File and ProTaper with or without chloroform in the removal of gutta-percha during retreatment of mandibular premolars.

**Materials and Methods:**

Sixty mandibular premolars with one canal, and curvatures less than 30 degrees were used in this experimental study. They were instrumented with K-files and laterally obturated with condensed gutta-percha using AH26 as the sealer and were stored in 100% humidity at 37°C for 2 weeks. The teeth were randomly divided into four groups of 15 teeth each. Removal of gutta-percha was performed with H-File and ProTaper. All techniques were used with or without chloroform. The teeth were split longitudinally and the area of remaining gutta-percha/sealer on the root canal wall was explored under stereomicroscope. Retreatment time duration was also recorded for each sample. Data were analyzed statistically by Two-way ANOVA, t-test and Tukey’s.

**Results:**

In all groups, no significant difference was found in remaining gutta-percha and sealer with or without using chloroform, but chloroform shortened the time of retreatment. ProTaper left significantly less remaining filling materials than H-File (P<0.05). Retreatment time was significantly different between the studied groups (P<0.001).

**Conclusion:**

ProTaper Ni-Ti instruments proved to be more efficient and time-saving devices for removal of gutta-percha compared to H-File in canals with no or slight curvature.

## 1. Introduction

The success of nonsurgical root canal retreatment highly depends on removal of previous root filling material, bacteria and necrotic tissue [1]. Different methods have been proposed to gain this goal such as manual or rotary instrumentation, solvents and ultrasonics [1, 3]. Use of hand files with or without solvent is a commonly used technique. Chloroform is classified as a group 2B carcinogen by International Agency for Research of Cancer [4]. Despite the concerns about chloroform, this solvent is still the most widely used solvent [3]. Also its efficacy in root canal retreatment has been studied previously [5-8]. Some suggest that chloroform can demonstrate adverse effects on cleanliness of canal wall [8, 9] but this is still a matter of controversy [5].

The ability of different types of Nickel-Titanium rotary files have been investigated in different studies [3, 9-12]. Findings in regard to the efficacy of these systems compared to hand files are controversial [2, 9, 11-13].

ProTaper D series, containing three flexible instruments, are designed for root filling material removal from different thirds of the canal. They should each work at special torque and speed according to the manufacturer in electric motor controllers [14].

Since rotary instruments necessitate special education and equipment for proper operation hand files are still common use among dentists.

This study was designed to compare the effectiveness of H-File and ProTaper with or without chloroform solvent in removing gutta-percha from root treated human extracted mandibular premolars. The time taken to remove gutta-percha was also recorded and evaluated.

##  2. Material and Methods

For this experimental study 60 single-canalled mandibular premolars which were extracted for periodontal reasons were selected. The inclusion criteria were as follows: fully formed apices, no sign of internal/external resorption, verified radiographically/apical patency with K-file #10 (Dentsply, Maillefer, Ballaigues, Switzerland), root curvature less than 30◦ according to Schnieder criteria [15] and a tooth length of 21-23 mm.

Working length was established 1 mm short from the point a#10 K-file was visualized at the apex. Root canal treatment was accomplished using step-back technique, with MAF (Master Apical File) equal to #30 K-file (Dentsply, Maillefer, Ballaigues, Switzerland). Each subsequent instrument was withdrawn 1 mm up to size # 60. Canals were flushed with 5 mL 5.25% NaOCl, delivered with a 27 gauge needle, between each instrument. At the end of canal preparation smear layer was removed with 2 mL of 17 % EDTA and 2 mL 5.25% NaOCl followed by 2 mL normal saline. Canals were dried with #30 paper points (GAPADENT CO. Tianjin, China). Master cone #30 (GAPADENT CO. Tianjin, China) was placed at the working length and lateral condensation was accomplished using # 15 lateral cones and AH26 (Dentsply, Detry, Konstanz, Germany) as sealer. Access cavities were sealed temporarily with Coltosol (Ariadent, Coltosol, Iran) and teeth were incubated at 37◦C/100% humidity for 2 weeks. After that period the samples were randomly divided into 4 groups.

Retreatment procedure:

Group A: The #3 and #2 GG (Gates-Glidden) drills (Dentsply, Maillefer, Ballaigues, Switzerland) were used in a crown down technique to remove gutta-percha from the coronal part of the canal. Exactly 0.2 mL chloroform (Kimia, Tehran, Iran) was placed in the space prepared by GG drills. After 2 minutes a #15 H-file (Dentsply, Maillefer, Ballaigues, Switzerland) was introduced into the canal till it reached the working length. In order to remove the gutta-percha and sealer, canals were instrumented up to size 40. The solvent was refreshed when needed. Retreatment was deemed complete when no more filling materials or sealer was seen on the last instrument.

Group B: The specimens were treated the same as group A except that no solvent was used in this group.

Group C: ProTaper universal retreatment instruments were used in a crown-down manner as stated by the manufacturer in this group. After removal of the coronal third gutta-percha, 0.2 mL chloroform was placed in the reservoir space prepared by D1 and enough time was given to soften the gutta-percha. The softened gutta-percha was removed by D2 and D3 with the last instrument reaching the working length. The solvent was refreshed between D2 and D3.

Group D: The teeth in this group went through the same procedure as group C except for the use of the solvent.

It should be mentioned that all rotary instruments were used with an electric motor controller (ENDO-MATE, NSK, Japan). Torque and speed were set according to the manufacturer. Canals were irrigated with 0.5 mL 2.5% NaOCl between each instrument in all groups. The time required for retreatment in each group was recorded by a stop watch.

Remnant Evaluation

Two longitudinal grooves were made in the buccal and lingual aspects of samples with a diamond disc without entering the canal space. The teeth were sectioned longitudinally with a chisel. They were then inspected visually and the root half with more filling remnants was inspected under a stereomicroscope (Olympus, SZX9, Tokyo, Japan) with ×25 magnification attached to a Pentium V computer. Images were made of each half. The percentage of the area of the canal to the total area covered by sealer and gutta-percha were measured by Auto CAD 2007. The evaluator was blinded to the group assignment.

Statistical analysis was performed by means of Two-way ANOVA, t-test and Tukey’s test. Level of significance was set at P=0.05.

## 3. Results

All the retreatment cleansing techniques left some filling material inside the root canal. Two-way ANOVA showed no significant differences in remaining gutta-percha and sealer with or without chloroform between all groups.

Chloroform had no significant effect on remaining gutta-percha and sealer, but the two instrumentation techniques (manual or rotary) were significantly different.

**Figure 1.**
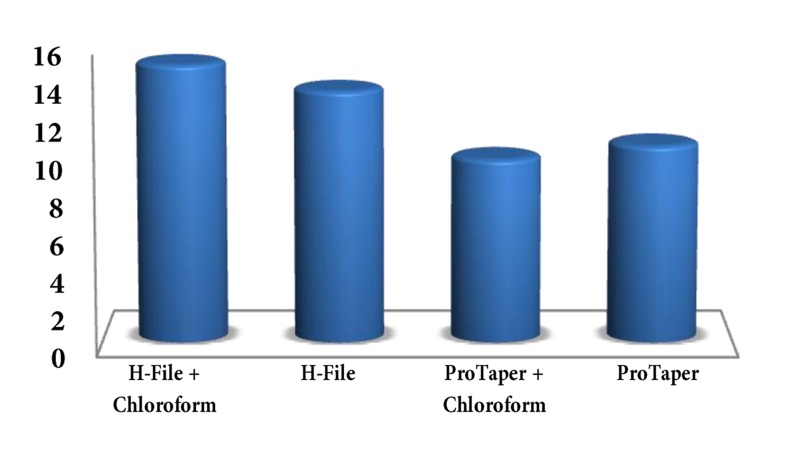
Remaining filling material in the canals (expressed as percentage of area) for each technique

T-test showed that the mean ratio of remaining filling material (mean ± SD) in the root canal was less with ProTaper compared with Hedstrom; the difference was statistically significant (P=0.021) (Figure 1).

**Figure 2.**
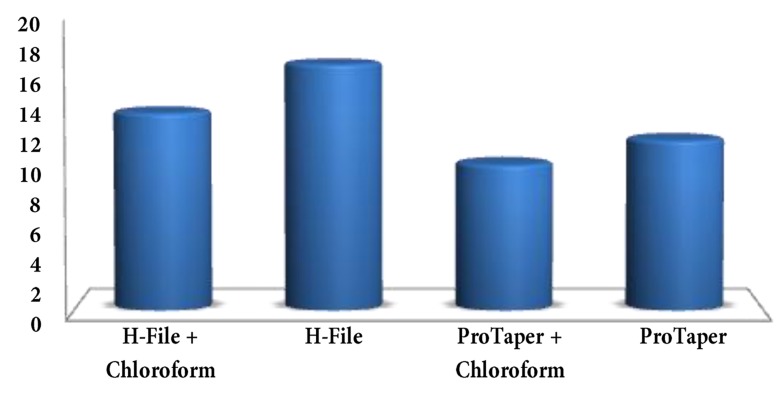
Mean time required (min) to remove filling material

There was significant difference between time required for retreatment among four groups (P<0.0001) (Figure 2) (multiple comparison, Table 1).

Table 1. Multiple comparisons of time taken to remove gutta-percha (the Tukey’s test, P=0.05)

**Table 1:** Multiple comparisons of time taken to remove gutta-percha (the Tukey’s test, P=0.05)

Multiple comparisons of time		P-value
**H-file with Chloroform**	H-file without chloroform	0.002
	ProTaper with chloroform	0.001
	ProTaper without chloroform	0.156
**H-file without Chloroform**	ProTaper with chloroform	0.000
	ProTaper without chloroform	0.000
**ProTaper with chloroform**	ProTaper without chloroform	0.239

Time taken for retreatment was shortest with ProTaper with chloroform followed by ProTaper without chloroform, H-File + chloroform and finally H-File without chloroform.

## 4. Discussion

Adequate removal of previous root filling materials plays a major role in the success of orthograde retreatment. In this study, like most previous studies [1, 2, 10], remnant fillings were found on root canal walls after root cleavage in all groups. Longitudinal cleavage of root in a buccolingual direction is a practical method to evaluate the effectiveness of the retreatment method [1, 2, 5, 19, 20] and unlike radiographic images [13, 21] provides a three dimensional view of the canal. The important point in this method as mentioned by Takahishi et al. is that the chisel should not touch the root canal walls [20].

Among different retreatment methods; retreatment rotary instruments have become more attractive [13, 19, 20, 22-25]. In this study ProTaper D series specified for retreatment purposes were compared with H-Files which were traditionally used for root canal retreatment. Although some studies have found no significant difference among ProTaper D and hand files efficacy in removing gutta-percha remnants on canal walls [20, 26], Unal et al. found K-files and H-files to be more effective in removing filling material than ProTaper and R-Endo instruments in curved canals [13]. In our study, ProTaper retreatment without solvent visibly showed less filling remnants. The slight curvature of the specimens might have allowed better performance of the D series instruments (D1, D2, D3) with tapers equal to (9%, 8% and 7% respectively). They are more likely to contact the root canal walls and remove filling remnants compare to 2% tapered H-Files. Gu et al. suggested that better performance of ProTaper D series in straight canals was due to the progressive taper and length of these files [9]. They mentioned that this design may result in not only removal of gutta-percha but also cut the superficial layer of dentin. Chloroform is considered a common solvent which has been used in many studies [7, 8, 20, 23, 24, 27]. In terms of root canal wall cleanliness, we found that solvent did not play significant role; this concurred with Takahashi et al. [20]. Also, in a recent study, Dadresanfar et al. [18] showed that solvent application had adverse effect on retreatment ability of Mtwo R instruments. Although Horvath et al. found less filling remnants in their non-solvent group, they only compared hand files with or without solvent [8]. The use of solvent has reduced the time needed for retreatment in some studies [11, 23, 28]. In the present study, the shortest retreatment time was in the ProTaper + solvent group. It seems that the heat generated by rotary instruments helps the solvent to plasticize the gutta-percha and eases the penetration of rotary instruments into the gutta-percha mass. Bramante and Betti believe softened gutta-percha is less resistant and easier to be penetrated [28]. Since antibacterial effect of chloroform has been proved in a study by Edgar et al. [6] and it shortens the time required for retreatment by ProTaper D series the authors assume that its use might be beneficial during retreatment procedure.

Since root curvature plays important role on the efficacy of root canal instrumentation, further investigations on severely curved roots is suggested.

## 5. Conclusion

Under the conditions of the present study, ProTaper D retreatment series with chloroform compare to hand instruments performed faster and more effective root canal retreatment in straight root canals. However, complete root canal cleanliness was not found in the studied groups.
